# Maternal Fat Feeding Augments Offspring Nephron Endowment in Mice

**DOI:** 10.1371/journal.pone.0161578

**Published:** 2016-08-22

**Authors:** Stacey Hokke, Victor G. Puelles, James A. Armitage, Karen Fong, John F. Bertram, Luise A. Cullen-McEwen

**Affiliations:** 1 Development and Stem Cells Program, Monash Biomedicine Discovery Institute, and Department of Anatomy and Developmental Biology, Monash University, Melbourne, Victoria, Australia; 2 School of Medicine (Optometry), Deakin University, Waurn Ponds, Victoria, Australia; The University of Manchester, UNITED KINGDOM

## Abstract

Increasing consumption of a high fat 'Western' diet has led to a growing number of pregnancies complicated by maternal obesity. Maternal overnutrition and obesity have health implications for offspring, yet little is known about their effects on offspring kidney development and renal function. Female C57Bl6 mice were fed a high fat diet (HFD, 21% fat) or matched normal fat diet (NFD, 6% fat) for 6 weeks prior to pregnancy and throughout gestation and lactation. HFD dams were overweight and glucose intolerant prior to mating but not in late gestation. Offspring of NFD and HFD dams had similar body weights at embryonic day (E)15.5, E18.5 and at postnatal day (PN)21. HFD offspring had normal ureteric tree development and nephron number at E15.5. However, using unbiased stereology, kidneys of HFD offspring were found to have 20–25% more nephrons than offspring of NFD dams at E18.5 and PN21. Offspring of HFD dams with body weight and glucose profiles similar to NFD dams prior to pregnancy also had an elevated nephron endowment. At 9 months of age, adult offspring of HFD dams displayed mild fasting hyperglycaemia but similar body weights to NFD offspring. Renal function and morphology, measured by transcutaneous clearance of FITC-sinistrin and stereology respectively, were normal. This study demonstrates that maternal fat feeding augments offspring nephron endowment with no long-term consequences for offspring renal health. Future studies assessing the effects of a chronic stressor on adult mice with augmented nephron number are warranted, as are studies investigating the molecular mechanisms that result in high nephron endowment.

## Introduction

It is well established that the metabolic health of an individual is shaped by the nutritional status of the mother. Adverse *in utero* and early postnatal environment conditions, as a consequence of poor maternal nutrition, can programme susceptibility in offspring to cardiovascular and metabolic disease in adulthood [1, 2]. Poor maternal nutrition may also alter kidney development and program renal injury and hypertension [3]. Brenner and colleagues proposed that a congenital or programmed deficit in nephron number may explain the increased risk of hypertension and renal disease in people of low birth weight, a surrogate marker for an adverse *in utero* environment [4]. In the context of maternal undernutrition, experimental models of maternal caloric restriction and maternal low protein diet have reported a nephron deficit in low birth weight offspring that subsequently develop hypertension and renal dysfunction in adulthood [5–7]. However, despite the current obesity epidemic, few studies have assessed kidney development and renal function in offspring exposed to maternal overnutrition and maternal obesity.

Obesity is attributed to physical inactivity, urbanisation, and the consumption of highly palatable, energy dense foods, resulting in an imbalance in the number of calories consumed and expended. The prevalence of obesity has doubled in the past 20 years in Australia, the United States, and other Western countries [8, 9] and it is estimated that over 50% of women in the Western world enter pregnancy either overweight or obese [10]. Maternal obesity is associated with a myriad of adverse pregnancy outcomes including congenital malformations, pre-eclampsia, gestational diabetes, macrosomia and birth weight above the 90^th^ centile [11, 12]. Maternal obesity and overnutrition also have long-term consequences for offspring health and are associated with the programming of obesity, diabetes, hypertension and other features of metabolic syndrome [13–15], as seen in offspring subject to nutrient deprivation during development.

Given the paucity of studies in this field and the increasing prevalence of obesity in pregnancy, this study aimed to investigate the effects of maternal overnutrition and obesity on offspring kidney development and renal function. Female C57Bl6 mice were fed a ‘Western style’ high fat diet to induce metabolic alterations and obesity prior to pregnancy. Ureteric branching morphogenesis and nephron endowment were assessed in offspring of high fat and normal fat fed dams to determine the effect of maternal overnutrition on kidney development. In addition, renal function and metabolic parameters were assessed in adult offspring to investigate the long-term consequences of exposure to excessive nutrition during gestation and lactation.

## Materials and Methods

### Animals

All animal handling and experimental protocols were carried out in accordance with the *Australian Code for the Care and Use of Animals for Scientific Purposes*, National Health and Medical Research Council of Australia. The protocol was approved by the Animal Ethics Committee of Monash University. Five week old female C57BL6/J mice (Monash Animal Research Platform) were housed in a controlled environment (12-hour light-dark cycle) with *ad libitum* access to water and food. Mice were fed a high fat diet (HFD; 21% fat w/w; SF00-219 semi-pure rodent diet; Specialty Feeds, Glen Forrest, WA, Australia) or a normal fat diet (designed by Specialty Feeds as a control diet for the HFD) (NFD; 6% fat w/w; SF04-057 semi-pure rodent diet; Specialty Feeds, Glen Forrest, WA, Australia) (**Table 1**). Mice were maintained on their respective diet for 6 weeks prior to pregnancy and throughout gestation and lactation until postnatal day (PN) 21.

**Table 1:** Nutritional parameters of the NFD and HFD.

Calculated Nutritional Parameters	NFD	HFD
Protein	19%	19%
Fat	6%	21%
Crude Fibre	4.7%	4.7%
Adequate Dietary Fibre	4.7%	4.7%
Digestible Energy	16.1 MJ/kg	19.4 MJ/kg
% Total digestible energy from lipids	14%	40%
% Total digestible energy from protein	21%	17%

### Maternal parameters

Female mice were weighed weekly (NFD *n* = 27, HFD *n* = 31) and energy intake was monitored twice weekly prior to conception (NFD *n* = 10, HFD *n* = 10). Glucose tolerance was assessed in all mice 6 weeks after commencement of the diet (NFD *n* = 27, HFD *n* = 31). Mice were administered a bolus of glucose (2 g/kg; i.p) following a 6 hour fast, blood was sampled from the tail vein at 0, 30, 60, 90 and 120 minutes and blood glucose concentrations were measured on a glucometer (Accu-chek Mobile Blood Glucose Monitor; Roche Diagnostics, Castle Hill, NSW, Australia). The glucose area under the curve (AUC) was calculated from the glucose tolerance curve. Body composition was measured at 6 weeks post diet by dual energy X-ray absorptiometry (DEXA; PIXImus2, Lunar, Madison, WI, USA) (NFD *n* = 20, HFD *n* = 22).

Six weeks following commencement of the diet, female mice were mated overnight with male C57Bl/6J mice. The presence of a vaginal plug confirmed that mating had taken place and was designated embryonic day (E) 0.5 of pregnancy. To circumvent the confounding effect of male high fat feeding, males were interchanged between cages of HFD and NFD females every 48 hours. Maternal weight was measured at E0.5 (NFD *n* = 27, HFD *n* = 31), E15.5 (NFD *n* = 8, HFD *n* = 8), E18.5 (NFD *n* = 7, HFD *n* = 10) and PN21 (NFD *n* = 12, HFD *n* = 13). Maternal energy intake was monitored between E0.5 and E18.5 (NFD *n* = 7, HFD *n* = 7). Glucose tolerance tests were performed in dams at E15.5 (NFD *n* = 8, HFD *n* = 8), E18.5 (NFD *n* = 7, HFD *n* = 10) and PN21 (NFD *n* = 12, HFD *n* = 13), as described above.

### Tissue collection to assess kidney development

Offspring were collected at E15.5, E18.5 and PN21 to assess kidney development. For embryonic tissue collection, dams were anaesthetised with isoflurane and exsanguinated by left ventricular puncture. Embryos were removed and placentae and embryos were weighed (E15.5: NFD *n* **=** 58 pups from 8 litters, HFD *n* **=** 61 pups from 8 litters. E18.5: NFD *n* **=** 64 pups from 7 litters, HFD *n* **=** 84 pups from 10 litters). Maternal and fetal plasma was collected and stored at -80°C until required. Note that blood samples were collected from mice following a 6 hour fast and 2 hour glucose tolerance test. Metanephroi were removed and fixed in 4% paraformaldehyde in phosphate buffered saline (PBS). PN21 offspring (NFD *n* **=** 76 pups from 12 litters, HFD *n* **=** 73 pups from 13 litters) were anaesthetised, perfusion fixed with paraformaldehyde-glutaraldehyde solution (Karnovsky’s Fixative) and the kidneys collected.

### Ureteric tree development at E15.5 and E18.5

Ureteric branching morphogenesis is a major feature of metanephric kidney development. Ureteric tree development was assessed at E15.5 by Optical Projection Tomography (OPT) as previously described [16, 17]. E15.5 kidneys (NFD *n* = 12 kidneys from 6 litters, HFD *n* = 12 kidneys from 6 litters) were wholemount fluorescently immunostained for TROP-2 to localise the ureteric epithelium (mouse TROP-2 polyclonal Goat IgG; R&D systems, Minneapolis, MN, USA). Kidneys were optically cleared and imaged in a Skyscan Bioptonics 3001 OPT scanner (Bioptonics, Edinburgh, UK). OPT tomographic data were reconstructed using N-Recon software (SkyScan, Kontich, Belgium) and visualised and rendered using Drishti software (ANUSF VizLab, Canberra, ACT, Australia). Quantitative assessment of the ureteric tree was performed using Tree Surveyor software [17] from which branch number, ureteric tip number, ureteric tree length and ureteric tree volume were automatically calculated.

Ureteric tree volume was assessed at E18.5 as previously described [18]. E18.5 kidneys (NFD *n* = 14 kidneys from 7 litters, HFD *n* = 15 kidneys from 8 litters) were processed to paraffin and exhaustively sectioned at 4 μm. Ten evenly spaced sections were histochemically stained with biotinylated Dolichos biflorus agglutinin (DBA; Sigma-Aldrich, Castle Hill, NSW, Australia) to localise ureteric duct epithelium, and the volume of ureteric tree was estimated using point counting with an orthogonal grid [18].

### Glomerular number at E15.5, E18.5 and PN21

Absolute nephron number was determined at E15.5 using wholemount kidneys previously scanned by OPT (NFD *n* = 12 kidneys from 6 litters, HFD *n* = 12 kidneys from 6 litters). Kidneys were transferred to methanol and PBS and the surrounding agarose carefully removed. Kidneys were processed to paraffin and serially sectioned at 4 μm. E15.5 sections were histochemically stained with the lectin peanut agglutinin (PNA; Sigma-Aldrich, Castle Hill, NSW, Australia) to localise the plasma membrane of glomerular podocytes and counterstained with haematoxylin. Sections were projected using a light microscope and all PNA-positive glomeruli were counted.

Total glomerular number was estimated at E18.5 (NFD *n* = 21 kidneys from 7 litters, HFD *n* = 17 kidneys from 8 litters) and PN21 (NFD *n* = 15 kidneys from 8 litters, HFD *n* = 24 kidneys from 13 litters) using an unbiased stereological method as previously described [19, 20]. Kidneys were processed to paraffin and exhaustively sectioned at 4 μm (E18.5) or 5 μm (PN21). Ten evenly spaced section pairs were systematically sampled and stained with PNA. Section pairs were used to estimate PNA-positive glomeruli using the physical disector/fractionator combination. Kidney volume was estimated using the Cavalieri Principle. Mean glomerular volume and total glomerular volume were estimated as described previously [21, 22].

### Plasma insulin and leptin analyses at E18.5

Maternal and fetal plasma insulin and leptin were analysed at E18.5. Blood samples were collected following a 6 hour fast and 2 hour glucose tolerance test (NFD *n* = 7, HFD *n* = 10). Samples were measured by commercial ELISA (Crystal Chem, Downers Grove, IL, USA) and run in duplicate. Fetal samples from the one litter were pooled.

### Metabolic function and renal function in adult offspring

A cohort of pups from NFD and HFD dams were weaned onto standard rodent chow at PN21 (Barastoc; Ridley AgriProducts, Pakenham, VIC, Australia) (NFD *n* = 40 offspring from 11 litters, HFD *n* = 48 offspring from 15 litters). Offspring were weighed weekly until 9 months of age. DEXA scans and glucose tolerance tests were performed in all offspring at 6 and 9 months of age. Renal function was assessed in all offspring at 6 and 9 months by the non-invasive transcutaneous measurement of FITC-sinistrin clearance (half-life; t_1/2_) in conscious mice [23, 24]. t_1/2_ is the rate constant of the single exponential elimination phase of the fluorescence-time curve, and represents renal clearance of FITC-sinistrin as a parameter of renal function [24]. An increased t_1/2_ indicates a reduction in the rate of glomerular filtration. Spot urine albumin/creatinine ratio was measured by ELISA in offspring at 9 months of age (Exocell, Philadelphia, PA, USA) (NFD *n* = 12 offspring from 8 litters, HFD *n* = 12 offspring from 9 litters). Blood samples were collected in 9 month old offspring following a 6 hour fast, and plasma insulin concentrations measured by commercial ELISA (Mercodia, Uppsala, Sweden) (NFD *n* = 16 offspring from 11 litters, HFD *n* = 23 offspring from 15 litters). Nine month old offspring were perfusion fixed with Karnovsky’s Fixative and the kidneys collected for analysis of total glomerular volume as described above (NFD *n* = 13 offspring from 8 litters, HFD *n* = 20 offspring from 12 litters).

### Statistical analysis

All data are expressed as mean ± SEM. Statistical analyses were performed with GraphPad Prism 6 (San Diego, CA, USA) and IBM SPSS Statistics 20 (Chicago, IL, USA) software. Maternal glucose tolerance tests were analysed by repeated measures ANOVA. Other maternal parameters (body weight, energy intake, fat content, glucose AUC, and plasma insulin and leptin) were analysed by independent samples t-test following normality test. Offspring parameters were analysed by two-way ANOVA with offspring sex and maternal diet as main effects, incorporating a mixed linear model to account for any intra-litter bias [16, 25]. Offspring body weight and glucose tolerance post-weaning were analysed by two-way repeated measures ANOVA (diet × sex × time). Multiple regression analysis was performed using STATA 13 (College Station, TX, USA). Throughout *n* refers to the number of dams or litters. Dams with fewer than four pups per litter were excluded from analysis. Note that litter size was not standardised after delivery, and litter size ranged from four to eight pups per litter. *P*<0.05 was considered statistically significant.

## Results

### High fat fed mice are overweight and glucose intolerant prior to pregnancy

**Fig 1:**
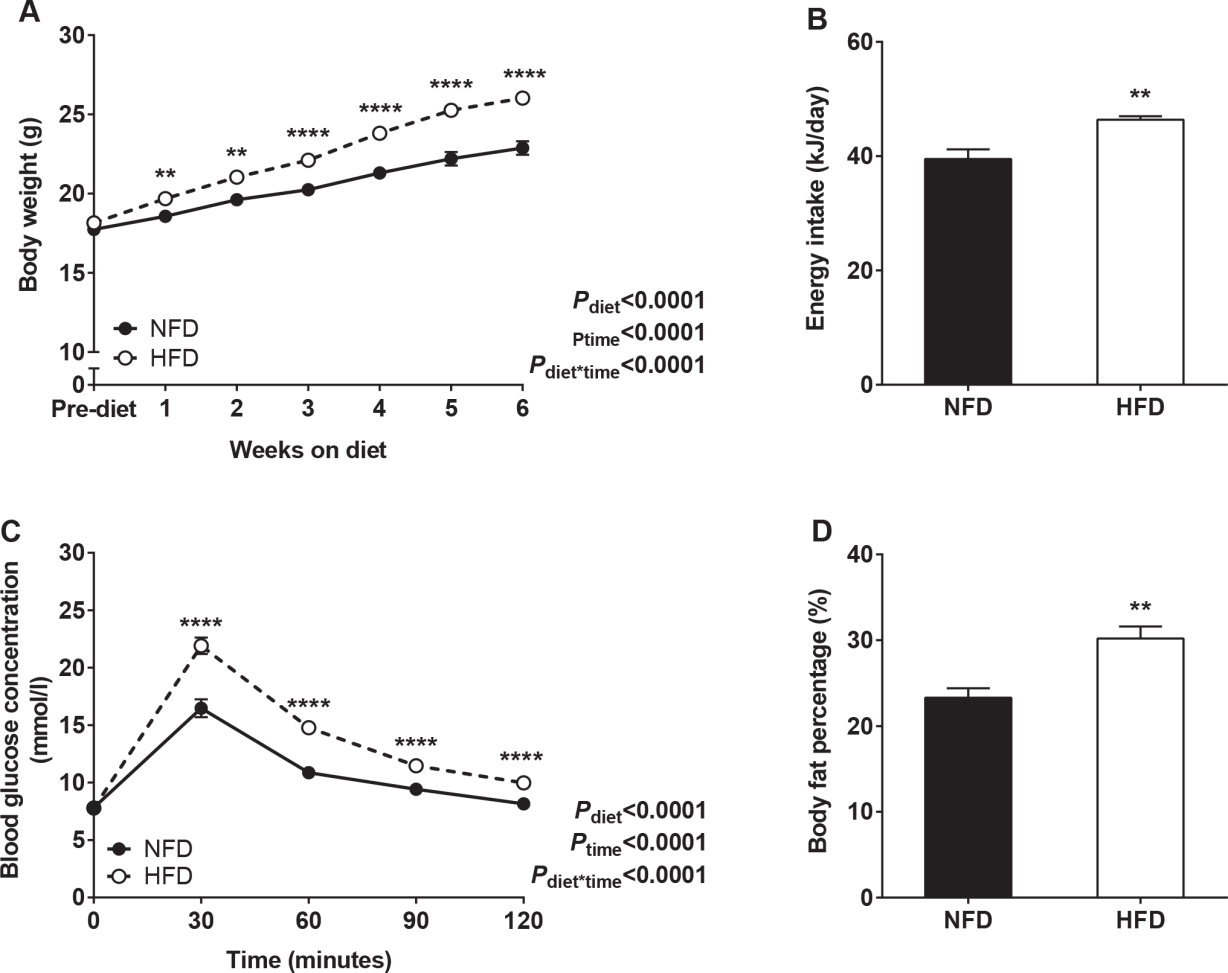
Maternal parameters prior to pregnancy. (A) Body weight and (B) energy intake across the 6 week pre-pregnancy period in NFD and HFD mice. (C) Glucose tolerance and (D) body fat percentage in NFD and HFD mice after 6 weeks on diet. **P<0.01, ****P<0.0001.

Mice fed the HFD gained significantly more weight and ingested more energy than mice fed the NFD during the 6 week pre-pregnancy period (**Fig 1A and 1B**). Following 6 weeks on diet, HFD mice were glucose intolerant (glucose AUC, mmol/l.min: NFD 1342.6±30.1; HFD 1711.8±39.0; *P*<0.0001) with normal fasting glucose levels, and had a greater fat content compared with NFD mice (**Fig 1C and 1D**).

### Increased body weight and glucose intolerance are not maintained in high fat fed mice in late gestation

Maternal parameters during pregnancy are presented in **Table 2**. HFD dams remained heavier at E0.5 and E15.5 compared with NFD dams, yet were of similar body weight at E18.5. Gestational weight gain was 15% less in HFD dams than in NFD dams. Glucose intolerance was not maintained in HFD dams at E15.5 or E18.5. Fasting glucose levels were comparable at E15.5 (NFD 7.8±0.3 mmol/l; HFD 8.3±0.4 mmol/l; *P* = 0.35), yet elevated in HFD dams at E18.5 (NFD 5.7±0.3 mmol/l; HFD 6.6±0.2 mmol/l; *P* = 0.03). Energy intake remained elevated in HFD dams compared with NFD dams during pregnancy. HFD dams were hyperinsulinaemic at E18.5 but displayed normal plasma leptin levels. There was no difference in body weight, fasting glucose or glucose tolerance between the two groups at PN21.

**Table 2:** Maternal body weight, glucose tolerance, energy intake and plasma insulin and leptin concentrations during pregnancy and lactation.

		NFD (*n*)	HFD (*n*)	*P*
**Body weight (g)**	**E0.5**	24.0 ± 0.4 (27)	26.1 ± 0.3 (31)	**0.002**
	**E15.5**	34.6 ± 0.6 (8)	37.8 ± 0.7 (8)	**0.002**
	**E18.5**	41.1 ± 1.0 (7)	40.3 ± 0.7 (10)	0.61
	**PN21**	30.8 ± 0.8 (12)	31.9 ± 0.8 (13)	0.36
**Pregnancy weight gain (E0.5-E18.5) (g)**		16.5 ± 0.5 (7)	14.0 ± 0.6 (10)	**0.006**
**Glucose AUC (mmol/l.min)**	**E15.5**	1663.1 ± 116.5 (8)	1824.4 ± 74.9 (8)	0.24
	**E18.5**	1352.4 ± 76.2 (7)	1482.9 ± 65.0 (10)	0.21
	**PN21**	1227.5 ± 121.8 (12)	1237.2 ± 104.5 (13)	0.95
**Pregnancy energy intake (E0.5-E18.5) (kJ/day)**		37.6 ± 1.3 (7)	49.1 ± 3.7 (7)	**0.02**
**Plasma insulin E18.5 (ng/ml)**		0.36 ± 0.03 (7)	0.58 ± 0.08 (10)	**0.04**
**Plasma leptin E18.5 (ng/ml)**		5.85 ± 0.52 (7)	6.61 ± 1.09 (10)	0.54

### Normal embryonic and early postnatal growth

Offspring parameters during gestation and at weaning are presented in **Table 3**. Maternal high fat feeding had no effect on litter size. Body weight was similar in offspring of HFD and NFD dams at E15.5, E18.5 and PN21. Placental weights were also similar in the two dietary groups at E15.5 and E18.5, with no difference in the ratio of fetal weight to placental weight at E15.5 or E18.5 (*P* = 0.95 and *P* = 0.33, respectively). There was no difference in fetal plasma insulin or leptin levels at E18.5. Body fat content was similar in offspring of NFD and HFD dams at PN21 (*P* = 0.26). Male offspring were significantly heavier than female offspring at PN21 (*P*<0.0001).

**Table 3:** Offspring litter size, body weight, placental weight and plasma insulin and leptin concentrations.

		NFD	HFD	*P*
**Litter size**	**E15.5**	8.1±0.4	8.9±0.5	0.27
	**E18.5**	9.1±0.4	8.4±0.3	0.16
	**PN21**	6.3±0.4	5.4±0.4	0.12
**Body weight**	**E15.5 (g)**	0.41 ± 0.01	0.40 ± 0.01	0.84
	**E18.5 (g)**	1.08 ± 0.02	1.11 ± 0.02	0.21
	**PN21 (g)**	9.8 ± 0.3	10.6 ± 0.4	0.15
**Placental weight**	**E15.5 (mg)**	109.2 ± 3.3	108.6 ± 3.9	0.91
	**E18.5 (mg)**	94.2 ± 6.1	105.2 ± 4.7	0.15
**Plasma insulin E18.5 (ng/ml)**		1.52 ± 0.20	1.93 ± 0.17	0.12
**Plasma leptin E18.5 (ng/ml)**		0.39 ± 0.08	0.42 ± 0.08	0.76

E15.5: NFD *n* **=** 58 pups from 8 litters, HFD *n* **=** 61 pups from 8 litters. E18.5: *n* **=** 64 pups from 7 litters, HFD *n* **=** 84 pups from 10 litters. PN21: NFD *n* **=** 76 pups from 12 litters, HFD *n* **=** 73 pups from 13 litters.

### Maternal high fat feeding increases offspring nephron endowment

**Fig 2:**
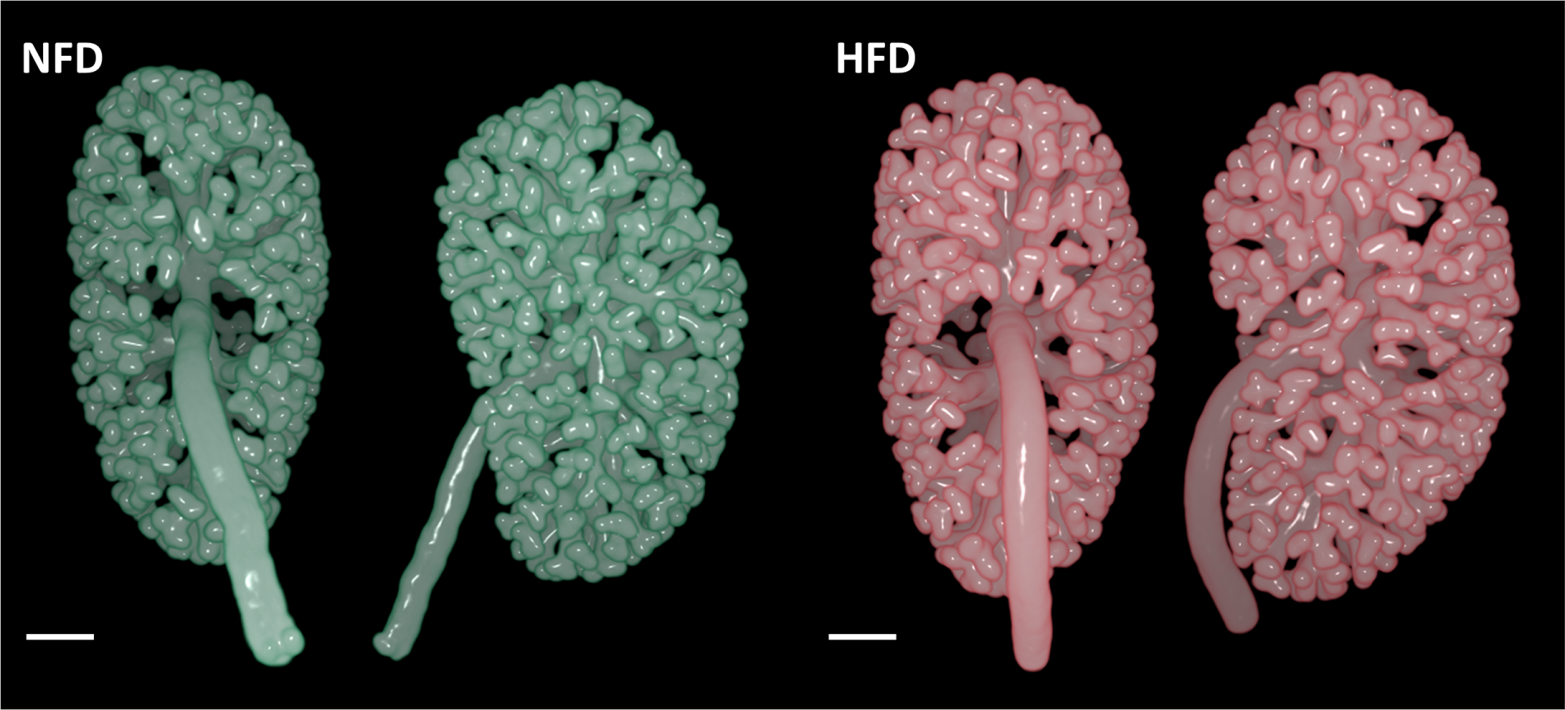
Representative OPT images of E15.5 kidneys from offspring of NFD and HFD dams. Ureteric epithelium rendered using Drishti software. Scale bar = 200 μm.

**Fig 3:**
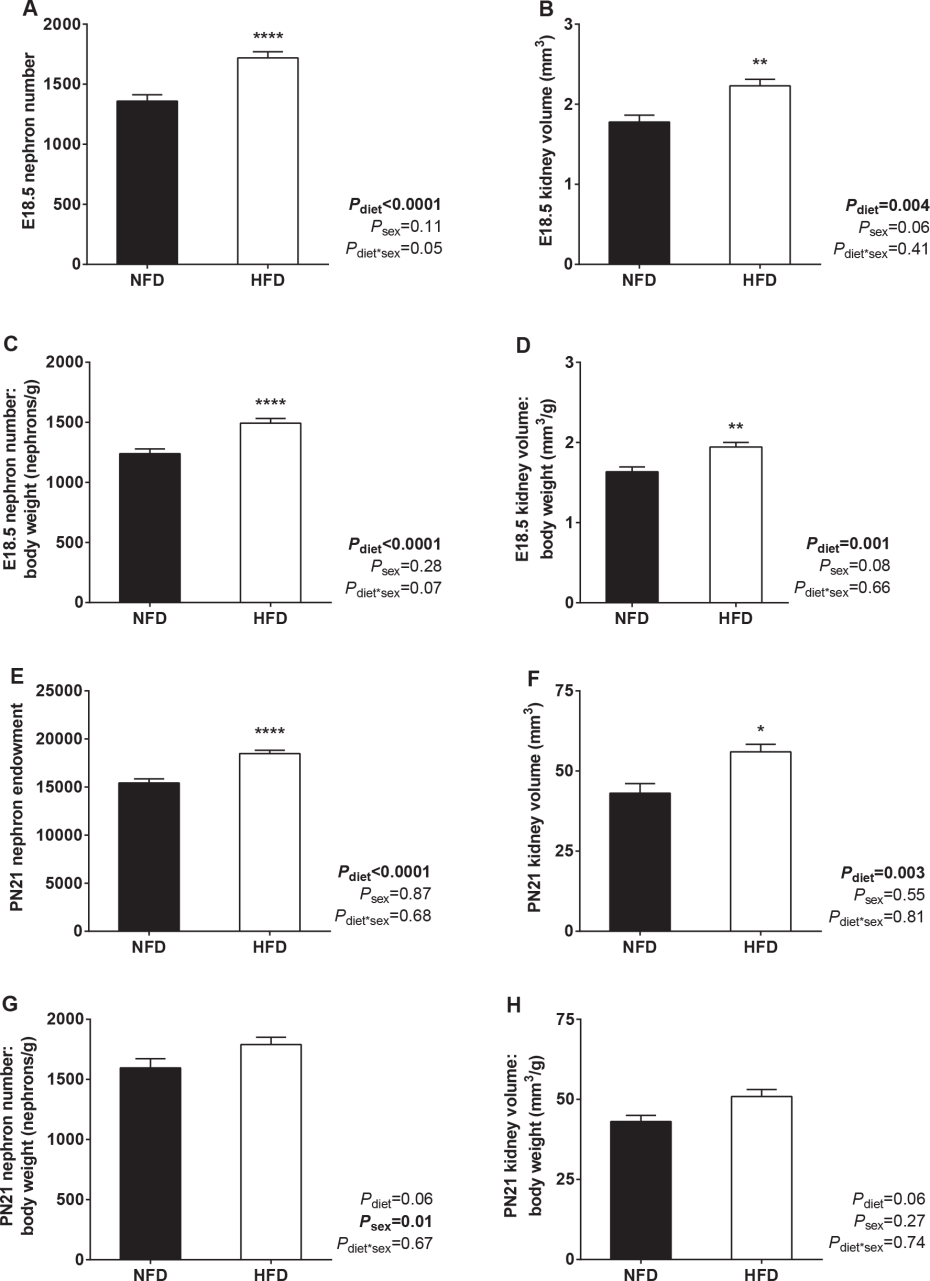
Offspring nephron number and kidney volume at E18.5 and PN21. (A) Nephron number, (B) kidney volume, (C) nephron number to body weight ratio and (D) kidney volume to body weight ratio in offspring of NFD and HFD dams at E18.5. (E) Nephron endowment, (F) kidney volume, (G) nephron number to body weight ratio and (H) kidney volume to body weight ratio in offspring of NFD and HFD dams at PN21. E18.5: NFD *n* = 21 kidneys from 7 litters, HFD *n* = 17 kidneys from 8 litters. PN21: NFD *n* = 15 kidneys from 8 litters, HFD *n* = 24 kidneys from 13 litters. *P<0.05, **P<0.01, ****P<0.0001.

**Fig 4:**
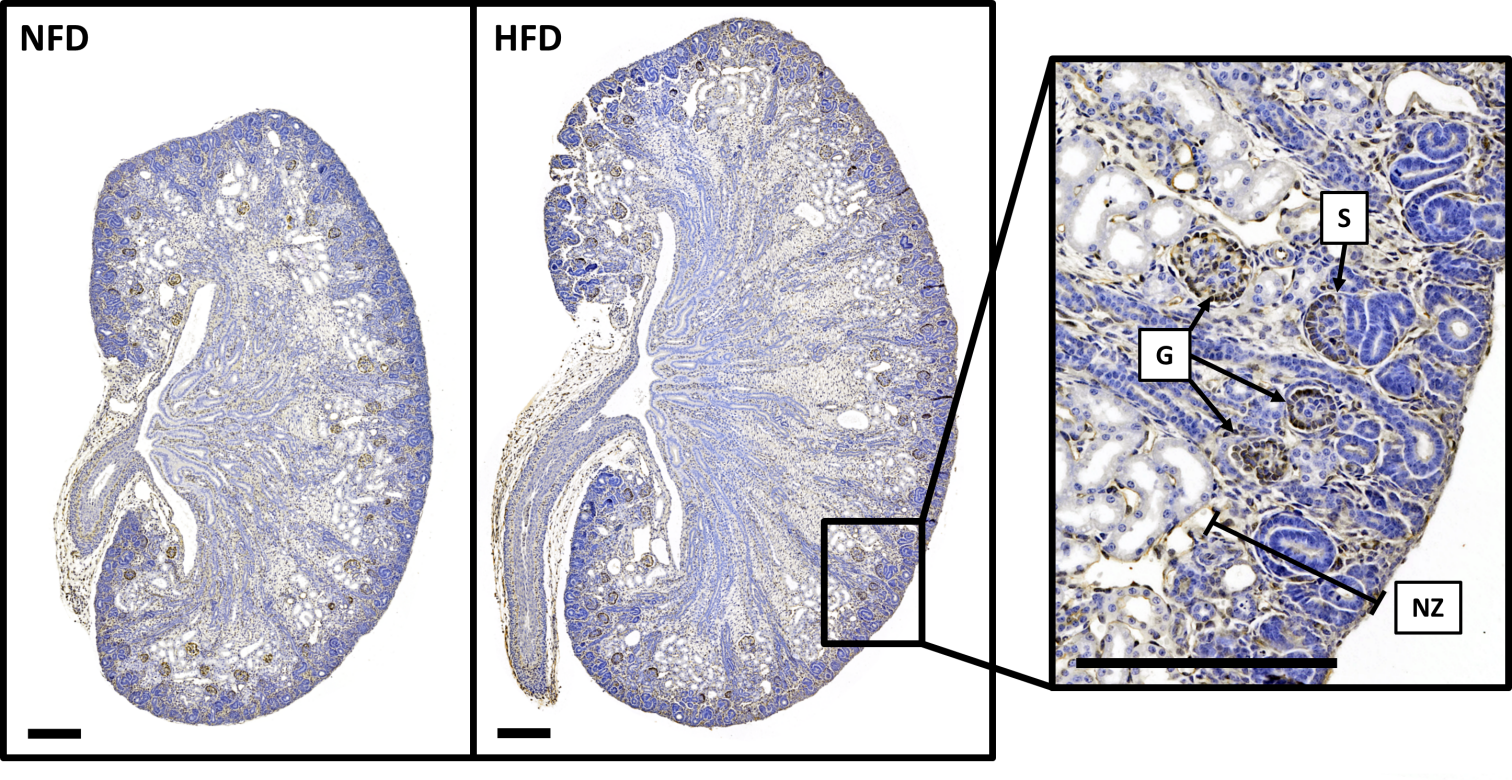
Representative images of sectioned E18.5 kidneys. Sections histochemically stained for PNA to identify glomeruli from the S-shaped stage to mature stage. Right panel highlights developing glomeruli at E18.5 at higher magnification. G = mature glomerulus, NZ = nephrogenic zone, S = S-shaped body. Scale bar = 200 μm.

Representative OPT images of ureteric trees at E15.5 are presented in **Fig 2.** There was no difference in ureteric tree development or nephron number in offspring of HFD and NFD dams at E15.5 (**Table 4**). Offspring ureteric tree volume was also similar at E18.5 (**Table 4**). However, E18.5 offspring of HFD dams had 26% more nephrons than offspring of NFD dams (**Fig 3A**). Kidney volume, nephron number to body weight ratio and kidney volume to body weight ratio were also higher in offspring of HFD dams at E18.5 (**Fig 3B, 3C and 3D**). Representative images of sectioned E18.5 kidneys are shown in **Fig 4**.

**Table 4:** Offspring ureteric tree development at E15.5 and E18.5.

		NFD	HFD	*P*_diet_
**E15.5**	**Ureteric tip number**	392 ± 15	409 ± 17	0.46
	**Ureteric branch points**	389 ± 15	406 ± 17	0.47
	**Ureteric tree length (μm)**	55,529 ± 2618	56,636 ± 2867	0.78
	**Ureteric tree volume (μm**^**3**^ **x 10**^**6**^**)**	92.0 ± 7.3	89.8 ± 8.0	0.85
	**Nephron number**	172 ± 12	179 ± 13	0.74
**E18.5**	**Ureteric tree volume (μm**^**3**^ **x 10**^**6**^**)**	90.0 ± 4.3	98.8 ± 4.3	0.21

E15.5: NFD *n* = 12 kidneys from 6 litters, HFD *n* = 12 kidneys from 6 litters. E18.5: NFD *n* = 14 kidneys from 7 litters, HFD *n* = 15 kidneys from 8 litters.

At PN21, kidneys of offspring of HFD dams contained 20% more nephrons than offspring of NFD dams (**Fig 3E**) and kidney volume was also higher in offspring of HFD dams at PN21 (**Fig 3F**). There was no statistical difference in nephron number to body weight ratio or kidney volume to body weight ratio between offspring of NFD and HFD dams at PN21 (**Fig 3G and 3H**). Mean glomerular volume was similar in offspring of NFD and HFD dams (V_glom_: NFD 0.64 ± 0.04 x10^-4^ mm^3^, HFD 0.65 ± 0.03 x10^-4^ mm^3^; *P* = 0.32). As expected, total glomerular volume was higher in offspring of HFD dams at PN21 (V_glom,kid_: NFD 0.97 ± 0.07 mm^3^, HFD 1.21± 0.06 mm^3^; *P* = 0.015). Total kidney weight (*P* = 0.07) and the kidney weight to body weight ratio (*P* = 0.18) were similar in the two groups at PN21.

### High fat fed dams with body weight and glucose profiles similar to normal fat fed dams have offspring with augmented nephron endowment

**Fig 5:**
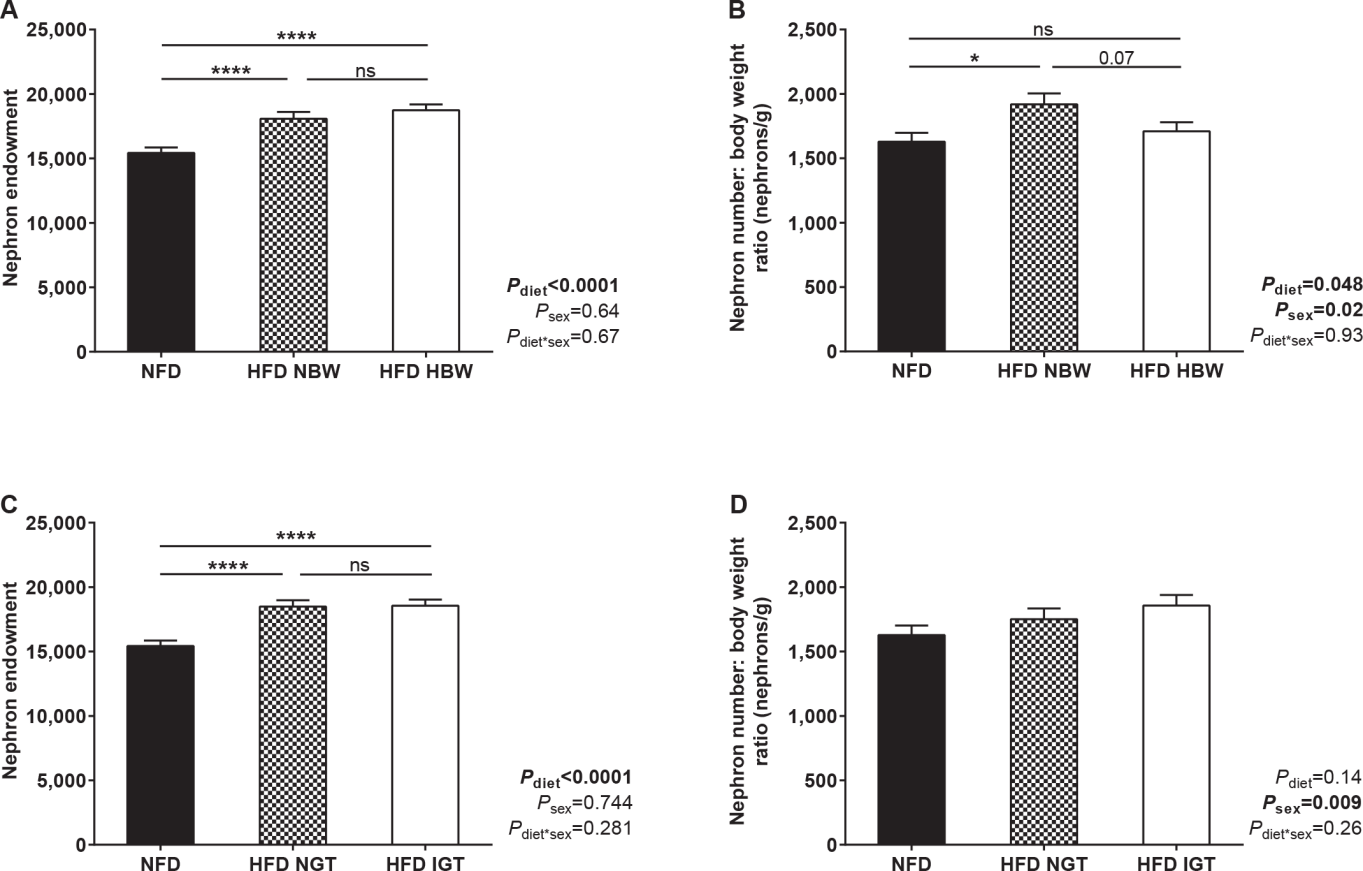
Defining the effect of maternal overweight and glucose intolerance prior to pregnancy on offspring nephron endowment at PN21. (A) Nephron endowment and (B) nephron endowment to body weight ratio in PN21 offspring of NFD, HFD NBW and HFD HBW dams. NFD *n* = 16 kidneys from 8 litters, HFD NBW *n* = 10 kidneys from 5 litters, HFD HBW *n* = 14 kidneys from 8 litters. (C) Nephron endowment and (D) nephron endowment to body weight ratio in PN21 offspring of NFD, HFD NGT and HFD IGT dams. NFD *n* = 16 kidneys from 8 litters, HFD NGT *n* = 12 kidneys from 7 litters, HFD IGT *n* = 12 kidneys from 6 litters. *P<0.05, ****P<0.0001, ns = not significant.

To help define the role of maternal fat feeding versus maternal obesity on offspring kidney development, offspring nephron endowment at PN21 was assessed based on their mother’s body weight prior to pregnancy. HFD dams were divided into two groups: those with pre-pregnancy body weight within one SD above the mean value for NFD dams (< 24.7g, abbreviated as HFD NBW, *n* = 5); and those with pre-pregnancy body weight greater than one SD above the mean value for NFD mice (> 24.7g, abbreviated as HFD HBW, *n* = 8). Nephron endowment was higher in offspring of both HFD NBW and HFD HBW dams than in offspring of NFD dams (**Fig 5A**). Offspring of HFD NBW dams had a greater nephron number to body weight ratio than offspring of NFD dams (**Fig 5B**).

Offspring nephron endowment was also assessed in dams based on their degree of glucose intolerance prior to pregnancy. HFD mice were divided into two groups: those with pre-pregnancy glucose AUC values within one SD above the mean value for NFD mice (< 1598 mmol/l.min, abbreviated as HFD NGT, *n* = 7); and those with pre-pregnancy glucose AUC values greater than one SD above the mean value for NFD mice (> 1598 mmol/l.min, abbreviated as HFD IGT, *n* = 8). As shown in **Fig 5C**, nephron endowment was similar in PN21 offspring of HFD NGT and HFD IGT dams, with both groups having approximately 20% more nephrons than offspring of NFD dams. There was no difference in nephron number to body weight ratio between the three groups (**Fig 5D**).

Multiple regression analysis was performed relating offspring nephron endowment at PN21 to offspring body weight at PN21, maternal body weight prior to pregnancy, maternal glucose tolerance prior to pregnancy as well as maternal diet (R2 = 0.63, P<0.0001). This revealed maternal diet to be the strongest predictor of nephron endowment (β = 0.70, P<0.0001) above that of offspring body weight (β = 0.24, P = 0.04), with no significant contribution from either maternal glucose levels or body weight prior to pregnancy.

### Normal body weight and fasting hyperglycaemia in adult offspring of high fat fed dams

**Fig 6:**
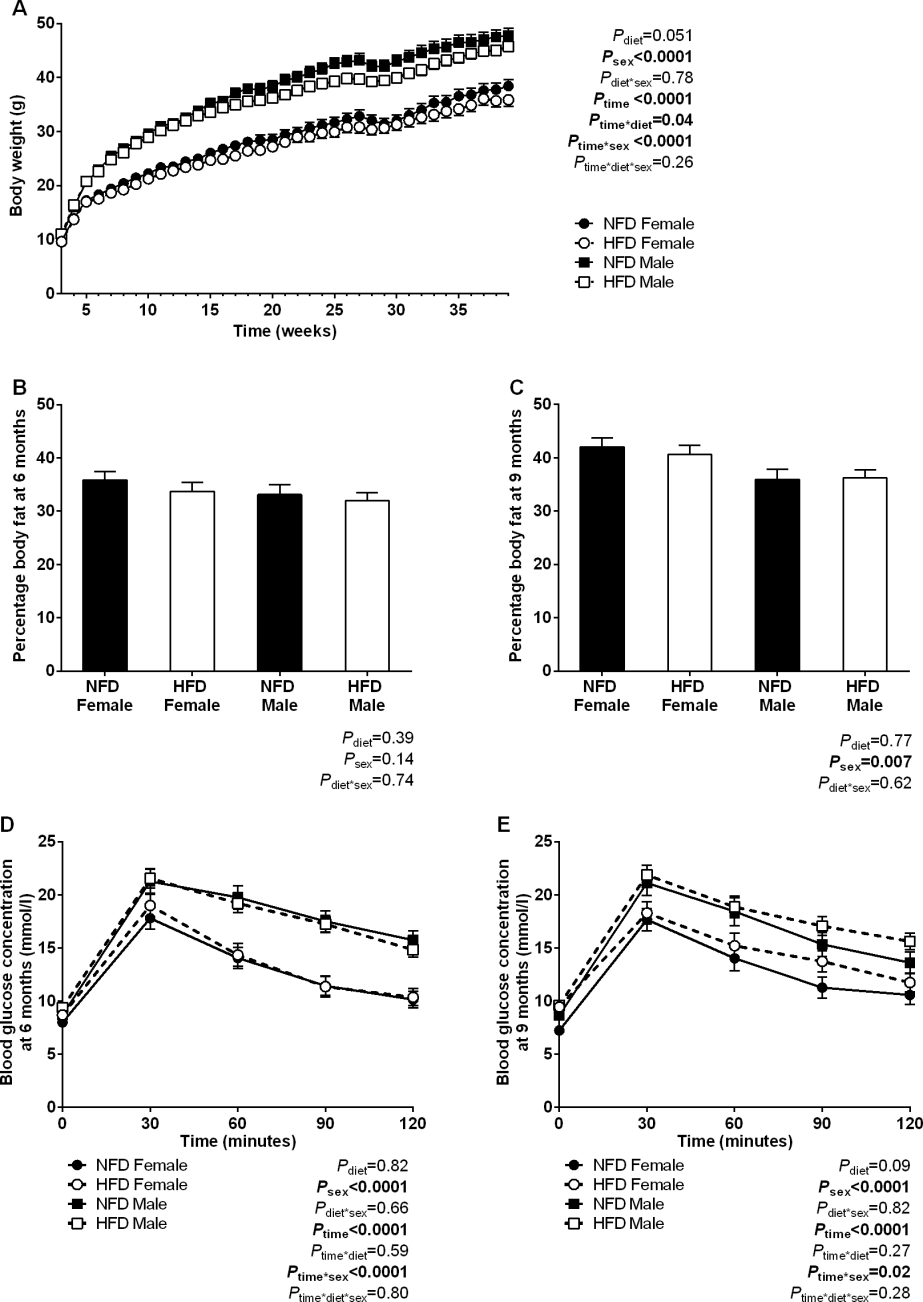
Body weight, fat content and glucose tolerance in adult offspring. (A) Body weight trajectories in offspring of NFD and HFD dams between PN21 and 9 months of age. (B) Body fat content and (C) glucose tolerance curves at 6 months and (D) body fat content and (E) glucose tolerance curves at 9 months in offspring of NFD and HFD dams. NFD *n* = 40 offspring from 11 litters, HFD *n* = 48 offspring from 15 litters.

Body weight trajectories in offspring of NFD and HFD dams from PN21 to 9 months of age are presented in **Fig 6A.** There was no statistical effect of maternal diet on offspring body weight trajectories (*P*_diet_ = 0.051) yet an interaction between maternal diet and time was observed (*P*_time*diet_ = 0.04), indicating reduced weight gain over time in adult offspring of HFD dams compared with NFD dams (**Fig 6A**). Body fat content and glucose tolerance were similar in offspring of NFD and HFD dams at 6 months of age (**Fig 6B and 6C**). However, at 9 months offspring of HFD dams displayed mild fasting hyperglycaemia (fasting glucose: NFD 8.0 ± 0.3 mmol/l, HFD IGT 9.5 ± 0.3 mmol/l; *P* = 0.003). Fasting insulin levels were similar in NFD and HFD offspring at 9 months of age (fasting plasma insulin: NFD 0.90±0.09 ng/ml, HFD 0.76±0.08 ng/ml; *P* = 0.26). There was no difference in body fat content or glucose tolerance in offspring of NFD and HFD dams (**Fig 6D and 6E**). Male offspring had elevated body weight, elevated glucose and insulin profiles and lower body fat content than female offspring.

### Normal renal function and glomerular volume in adult offspring of high fat fed dams

Maternal high fat feeding had no effect on offspring renal function at 6 or 9 months of age (**Table 5**). Urinary albumin/creatinine ratio was similar in offspring of NFD and HFD dams at 9 months. There was also no difference in total glomerular volume or kidney volume between the two dietary groups (**Table 5**). Male offspring had elevated t_1/2_ values and greater total glomerular volumes and kidney volumes than female offspring. Histological analysis of kidneys in offspring at 9 months of age showed no evidence of renal pathology.

**Table 5:** Renal function and renal morphology in adult offspring.

	NFD Female	HFD Female	NFD Male	HFD Male	*P*_*diet*_	*P*_*sex*_	*P*_diet*sex_
**Renal function (t**_**1/2**_**), 6 months**	11.7 ± 0.6	12.3 ± 0.6	16.1 ± 0.6	16.2 ± 0.5	0.60	**<0.0001**	0.70
**Renal function (t**_**1/2**_**), 9 months**	11.7 ± 0.5	12.4 ± 0.5	15.2 ± 0.6	15.6 ± 0.5	0.33	**<0.0001**	0.82
**Albumin/creatinine ratio (μg/mg)**	6.0 ± 1.4	5.9 ± 1.3	7.2 ± 1.3	7.7 ± 1.3	0.89	0.25	0.84
**Total glomerular volume, V**_**glom,kid**_ **(mm**^**3**^**)**	2.54 ± 0.21	2.31 ± 0.18	3.15 ± 0.22	2.87 ± 0.16	0.25	**0.02**	0.89
**Kidney volume (mm**^**3**^**)**	97.1 ± 6.9	99.1 ± 6.1	139.4 ± 7.5	136.4 ± 5.5	0.94	**<0.0001**	0.70

NFD *n* = 13–40 offspring from 8–11 litters, HFD *n* = 15–48 offspring from 12–15 litters.

## Discussion

An increasing number of women are entering pregnancy overweight or obese, yet few studies have assessed if and how maternal overnutrition and obesity can affect the developing kidney and subsequent renal health. To address this, maternal overnutrition and obesity were modelled by feeding female mice a HFD for 6 weeks prior to pregnancy and throughout gestation and lactation. We found that offspring exposed to maternal fat feeding had normal ureteric tree development and nephron number at E15.5. However, by E18.5 offspring of HFD dams had more nephrons than offspring of NFD dams, with this increase in nephron number maintained at PN21. When followed into adulthood, offspring of HFD dams were surprisingly neither overweight nor glucose intolerant, and exhibited normal renal function and renal morphology.

### Modelling maternal overnutrition and obesity

High fat feeding has been used extensively in rodents to model maternal obesity and overnutrition [26–31]. In the present study, mice fed a HFD for 6 weeks had greater energy intake and weight gain than NFD mice and, as expected, were glucose intolerant with increased body weight and adiposity at conception. However, this phenotype was not maintained in pregnancy. HFD dams continued to show an increase in energy intake during pregnancy yet gained less weight than NFD dams. Unfortunately, fat mass was not monitored in late gestation. Glucose tolerance was similar in HFD and NFD dams at E15.5 and E18.5, despite HFD dams displaying marked glucose intolerance prior to pregnancy as well as increased body weight at E15.5 and fasting hyperglycaemia and hyperinsulinaemia at E18.5. This indicates that mice had reached a threshold of obesity and glucose intolerance after 6 weeks of fat feeding that was not exacerbated by pregnancy.

The convergence of maternal weight in late gestation is consistent to that reported in some rodent studies of diet-induced obesity [29–31] but contradicts studies which showed that maternal weight is maintained at an increased level throughout pregnancy [26, 28, 32, 33]. Reports of glucose levels in pregnant rodents fed a HFD are similarly equivocal [26, 29, 32, 33]. Discrepancies in maternal metabolic phenotype in studies of fat feeding likely relate to differences in strain and species, the composition of control and high fat diets, and the length of time on diet prior to mating. It should be noted that in the present study maternal glucose tolerance tests were only performed on the day of embryo collection and, therefore, the developmental stage at which embryos were no longer exposed to elevated glucose levels is unknown.

### Offspring kidney development

Maternal overnutrition had no apparent effect on nephrogenesis in offspring at E15.5, yet higher nephron number was observed in HFD offspring at E18.5 when HFD dams were of comparable body weight and glucose tolerance to NFD dams. Moreover, offspring of HFD dams with body weight and glucose profiles similar to NFD dams prior to pregnancy had an elevated nephron endowment at PN21. This may suggest that augmented nephron endowment was due to certain constituents of the maternal HFD crossing to the offspring and promoting nephrogenesis, rather than as a direct effect of maternal obesity or hyperglycaemia. Additional studies that directly assess the contribution of maternal fat feeding and obesity are required, and should incorporate extra experimental groups and changing maternal diets at conception. It should be noted that the fat source differed between the diets in the present study (NFD fat sourced from canola oil, HFD fat sourced from ghee), which may also have contributed to the difference in nephron endowment. This is an important consideration given the marked difference in polyunsaturated fat content between these two fat sources, resulting in a deficiency in polyunsaturated fats in the HFD. Further studies assessing maternal and offspring lipid profiles are warranted.

Few studies have reported an increase in nephron endowment due to an environmental insult. Augmented nephron number has been reported in offspring of dams treated with retinoic acid and in models of maternal vitamin D deficiency and water restriction [34–36]. Of relevance to the present study is the increase in offspring nephron endowment due to postnatal hypernutrition reported by Boubred et al. [37]. Using a rat model of early postnatal overnutrition induced by a reduction of litter size at PN3, Boubred et al. [37] observed an acceleration in pup growth and a 20% increase in nephron endowment. While the authors did not examine the mechanisms promoting postnatal nephrogenesis, it was postulated that an increase in availability of energy, nutritional substrates and growth factors within the kidney likely contributed to the increase in nephron endowment. Similar mechanisms may also have played a part in the present study.

It is interesting that the stimulatory effects of maternal fat feeding on offspring nephrogenesis were observed at E18.5 but not at E15.5, and without an increase in ureteric tree development. Due to limitations in the spatial resolution of increasingly dense ureteric tips at E18.5, it was not feasible to assess ureteric branching morphogenesis by OPT at this time point. It was also not possible to measure ureteric tip number in kidney sections at E18.5, and thus the number of tips (and therefore the number of sites available for nephron induction) in HFD kidneys at E18.5 is unknown. While there was no statistical difference in ureteric tree volume at either embryonic time point, tree volume was slightly lower at E15.5 and slightly higher at E18.5 in HFD offspring, which may have contributed to the increased nephron number at E18.5. Alternatively, the increase in nephron number at E18.5 may reflect an increase in the number of nephrons formed in arcades. The marked increase in nephron formation between E15.5 and E18.5 may also be due to placental maturity and temporal changes in the transfer of nutritional substrates from the maternal to fetal circulation [33, 38, 39]. Future studies should aim to identify the underlying mechanisms of augmented nephrogenesis in a high fat environment, particularly the effect of maternal fat feeding on placental development, nutrient transfer and the molecular regulation of kidney development.

### Offspring growth and metabolic health

Maternal obesity and glucose intolerance are commonly associated with fetal overgrowth and neonatal adiposity. Rodent studies of pre-gestational high fat feeding have also reported heavier offspring at birth and at weaning [28, 30, 31, 33, 40], although increases in early offspring body weight are not universally reported [26, 41, 42]. In the present study, HFD offspring were of normal body weight at E15.5, E18.5 and at weaning. These findings are not unique in view of the variable effect of fat feeding on offspring growth in rodents, but do prove interesting considering the remarkable acceleration in kidney size and nephron endowment without an associated increase in body weight. It should be noted that litter size was not standardised after delivery in the present study. Although mean litter size was comparable between NFD and HFD dams and ranged from four and eight pups per litter after birth, variations in milk supply between and within dietary groups may exist.

Exposure to maternal fat feeding had surprisingly little effect on the metabolic health of offspring in adulthood and, if anything, offspring of HFD dams were underweight in the postnatal period. In contrast to the present study, studies of maternal fat feeding commencing prior to pregnancy and extending into lactation typically report a 10–20% increase in offspring weight by approximately 3 months of age compared with controls [30–32, 40, 43–47]. Furthermore, a recent meta-analysis of 53 studies in mice and rats by Lagisz et al. [48] identified a substantial positive effect of a maternal obesogenic diet on offspring weight in adult life. However, Lagisz et al. [48] also identified that dams fed a HFD with a low protein to non-protein ratio have smaller offspring than control offspring. The authors speculated that limitations in maternal protein intake via consumption of an obesogenic diet may modify the extent of developmental programming in offspring. When designing the present study we avoided the use of a standard chow diet as a control diet and endeavoured to match the NFD and HFD as much as possible. While both diets are matched gram for gram in regards to protein, the ratio of protein to non-protein content by calories is in fact lower in the HFD (NFD 0.26 vs. HFD 0.20). The relatively low protein content may therefore explain the lack of a programming effect in adult offspring exposed to maternal fat feeding, concerning body weight at least. However, with respect to the developing kidney it is unlikely that relative protein depletion in the HFD contributed to an augmented nephron endowment considering that maternal protein restriction is known to reduce nephron number [5, 49, 50]. Further assessment of offspring kidney development in studies that utilise high fat diets with appropriate protein caloric content are required.

Maternal fat feeding has been shown to program poor glycaemic control in offspring [51]. In the present study, HFD offspring had fasting hyperglycaemia at 9 months of age but not at 4 or 6 months. The increase in fasting blood glucose concentration over time may suggest an inability of HFD offspring to maintain glucose homeostasis with advancing age, as shown previously [31, 41]. While glucose tolerance was normal at 9 months of age, it is plausible that glucose intolerance may develop in older offspring. The presence of fasting hyperglycaemia despite normal body weight and body composition may suggest a primary pancreatic issue or skeletal insulin resistance not mediated by obesity. Further studies are required to assess glucose handling and pancreatic function in aged offspring.

### Offspring renal health

Studies assessing developmental programming of renal function and structure in maternal overnutrition are limited. Previously, Armitage et al. [52] found offspring of rats fed a HFD for 10 days prior to mating and throughout gestation and lactation were hypertensive at 6 months of age [53], yet with normal nephron number and glomerular volume. Interestingly, increased blood pressure was confined to female offspring. In a study by Jackson et al. [54], male offspring of rats fed a high fat, high fructose diet for 6 weeks prior to pregnancy, throughout gestation and lactation, had normal blood pressure and glomerular filtration rate (GFR) yet increased urinary albumin excretion, glomerulosclerosis and tubulointerstitial fibrosis by 4 months of age. Unfortunately, GFR and albuminuria were not reported by Armitage et al. [52] and nephron number and glomerular volume were not assessed by Jackson et al. [54].

Few studies have assessed the long-term consequences of elevated nephron endowment. Heterozygous null TGF-β2 mice show a 30–60% increase in nephron endowment but normal urine osmolality, albumin excretion, creatinine clearance and blood pressure at 4–6 months of age [55, 56]. As mentioned previously, Boubred et al. [37] reported a 20% increase in nephron endowment in rats exposed to early postnatal hypernutrition. This was associated with hypertension, increased protein excretion rate, glomerular hypertrophy and glomerulosclerosis in adult male offspring at 22 months of age. More studies of renal function and pathology in animals with abnormally high nephron endowment are required.

A high nephron endowment may also have a protective effect on renal function and glomerular morphology, as shown in heterozygous TGF-β2 mice following a chronic insult [55]. Exposure to a high salt diet raised mean arterial pressure in wild type mice, but not heterozygous TGF-β2 mice. The blunted arterial pressure response in mice with a high nephron endowment may have been due to a lower single nephron GFR, protecting mice from hyperfiltration-induced injury. The protective effect of a high nephron endowment was not observed in male offspring exposed to early postnatal hypernutrition [37]. This may be related to glomerular hypertrophy, but may also be due to the long-term effects that overnutrition during development has on metabolic health and function. In the study by Boubred et al. [37], adult offspring were slightly, but not significantly heavier despite considerable overgrowth in the early postnatal period. Adiposity and glucose tolerance were not measured, yet it is possible that postnatal overnutrition may have programmed features of metabolic syndrome in addition to hypertension that, in turn, may have been detrimental to renal function and structure.

Considering that HFD offspring in the present study had augmented nephron endowment and normal metabolic function in adulthood, it is not surprising that maternal overnutrition had no long-term effects on renal function or morphology. Further experiments incorporating exposure of adult offspring to a second insult, such as a high fat feeding challenge or high salt diet challenge, would be of interest. Such chronic stressors may reveal a protective effect of enhanced nephron endowment or, conversely, may reveal a reduced functional renal reserve in offspring exposed to maternal fat feeding.

### Concluding remarks

The present study has shown that a maternal high fat diet can result in higher nephron endowment in mouse offspring. Maternal fat feeding and overnutrition augmented offspring nephron number, even in HFD dams with weight and glucose profiles comparable to that of NFD dams prior to conception. As adults, offspring exposed to the maternal HFD had normal renal function and morphology and were not overweight or glucose intolerant. Considering that 9 month old mice are equivalent to middle age in humans, further studies should assess the metabolic and renal phenotype in aged offspring. Additional studies incorporating a high fat feeding challenge are also warranted as, despite an elevated nephron endowment, offspring of high fat fed dams may exhibit abnormal nephron structure and a reduced functional renal reserve that is only unmasked in response to a chronic stressor.
